# Time Course and Comparative Risk of Hypophosphatemia After Ferric Carboxymaltose versus Ferric Derisomaltose: A Systematic Review, Meta-analysis, and Clinical Cost Considerations

**DOI:** 10.1177/00185787261485528

**Published:** 2026-10-24

**Authors:** Vidhita Dangre, Mohd Rafiuddin, Krithika Sriram, Iqra Aslam Siddiqui, Hadia Fahim, Shanthakumar Sakthivel Murugan, Federica Copeta, Noor Ul Ain Fatima, Belinda Eze, Swostika Pokhrel

**Affiliations:** 1 School of Medicine, Xiamen University, Xiamen, China; 2 ESIC Medical College and Hospital, Sanathnagar, Hyderabad, India; 3 Flinders University, Adelaide, Australia; 4 DOW University of Health Sciences, Karachi, Pakistan; 5 Davao Medical School Foundation, Inc., Davao City, Philippines; 6 Royal College of Surgeons in Ireland, Dublin, Ireland; 7 Bahria University Health Sciences Campus, Karachi, Pakistan; 8 St George’s University Hospitals NHS Foundation Trust, London, UK; 9 College of Medical Sciences, Bharatpur, Nepal

**Keywords:** ferric carboxymaltose, ferric derisomaltose, hypophosphatemia, intravenous iron, iron deficiency anemia

## Abstract

**Objectives:**

To compare the risk of hypophosphatemia and associated outcomes following ferric carboxymaltose (FCM) versus ferric derisomaltose (FDI) in adults with iron deficiency anemia.

**Methods:**

We conducted a systematic review and meta-analysis of studies identified through database searches up to November 2025. Randomized and observational studies comparing FCM and FDI were included. The primary outcome was the incidence of hypophosphatemia within 35 to 42 days after infusion. Secondary outcomes included severe and persistent hypophosphatemia, patient-reported outcomes, and economic outcomes. Data from randomized trials were quantitatively pooled using a random-effects model to calculate risk ratios (RRs) with 95% confidence intervals (CIs), whereas findings from observational and economic studies were summarized narratively.

**Results:**

Twenty-one studies were included, comprising 3 randomized controlled trials (364 participants) and 18 observational or economic evaluation studies. In pooled randomized comparisons, FCM was associated with a higher risk of hypophosphatemia within 35 to 42 days than FDI (RR 8.37, 95% CI 5.10-13.75; *P* < .00001; *I*^2^ = 0%). Severe hypophosphatemia occurred in 11.3% of patients receiving FCM and was not observed with FDI. Persistent hypophosphatemia beyond 5 to 8 weeks was more frequent with FCM. Fatigue scores improved more with FDI. Economic evaluations reported greater quality-adjusted life years and lower healthcare costs with FDI.

**Conclusion:**

Ferric carboxymaltose is associated with a higher risk of hypophosphatemia compared with ferric derisomaltose. Ferric derisomaltose demonstrates a more favorable safety profile and consistent economic findings.

## Introduction

Iron deficiency anemia (IDA) is a highly prevalent global health challenge, frequently necessitating intravenous (IV) iron therapy when oral supplementation is ineffective, contraindicated, or limited by severe gastrointestinal adverse effects.^1,2^ In adults with chronic conditions, such as inflammatory bowel disease (IBD), gastrointestinal blood loss, chronic kidney disease (CKD), and postpartum anemia, IV iron rapidly restores diminished stores.^3,4^ Contemporary high-dose formulations, specifically ferric carboxymaltose (FCM) and ferric derisomaltose (FDI, also known as iron isomaltoside), represent a major therapeutic advance. They enable rapid, comprehensive iron repletion with only 1 or 2 infusions, even in cases of severe anemia while largely avoiding hypersensitivity risks associated with older high-molecular-weight dextran preparations.^3,5^

Despite their comparable hematologic efficacy, increasing clinical concerns have focused on the striking divergence in their metabolic safety profiles. FCM administration significantly impairs the proteolytic cleavage of fibroblast growth factor-23 (FGF23), leading to an acute elevation in circulating intact FGF23 (iFGF23).^6,7^ This pathological iFGF23 surge triggers a cascade leading to profound renal phosphate wasting, suppression of 1,25-dihydroxyvitamin D synthesis, subsequent hypocalcemia, and reactive secondary hyperparathyroidism.^6-8^ This FGF23-mediated pathophysiology predictably results in clinically relevant hypophosphatemia within 2 to 6 weeks post-infusion. This timeline aligns precisely with peak iFGF23 activity, thereby justifying a critical 35- to 42-day post-infusion window for accurate outcome assessment.

The clinical consequences of formulation-specific phosphate depletion are not merely transient biochemical anomalies. Moderate-to-severe hypophosphatemia can provoke life-threatening acute complications, including rhabdomyolysis, respiratory failure, and cardiac dysfunction.^8,9^ Furthermore, chronic phosphate depletion extending beyond 5 to 8 weeks carries a well-documented, severe risk of bone mineralization defects, culminating in osteomalacia and atypical insufficiency fractures.^8,10^ Crucially, the symptomatic burden of hypophosphatemia, manifesting as profound fatigue, muscle weakness, and bone pain, frequently undermines the quality-of-life benefits patients seek when treating the underlying anemia.^9,11^ Consequently, formulations with different hypophosphatemia risk profiles carry important economic and health-system implications that demand rigorous, head-to-head comparative evaluation.

While previous reviews have documented the occurrence of IV iron-induced hypophosphatemia,^12,13^ no systematic review has comprehensively integrated the time-specific risk of incident hypophosphatemia, its clinical severity, and its downstream economic consequences using strictly randomized head-to-head comparisons of FCM and FDI. Therefore, this meta-analysis aimed to evaluate the comparative incidence of hypophosphatemia within a biologically driven 35- to 42-day window following FCM versus FDI administration. As secondary objectives, we synthesized the risks of severe and persistent (≥5-8 weeks) hypophosphatemia, alongside symptomatic, economic, and quality-of-life outcomes, to provide a comprehensive safety and value comparison to assist in guiding clinical practice.

## Methods

### Protocol Reporting

We conducted and reported this systematic review and meta-analysis in strict adherence to the Cochrane Handbook for Systematic Reviews of Interventions and the Preferred Reporting Items for Systematic Reviews and Meta-Analyses (PRISMA) guidelines.^14,15^ Our study design was anchored by a predefined Population, Intervention, Comparison, and Outcome (PICO) framework (Figure S1). Because all analyses relied exclusively on previously published, publicly available data, this study did not require institutional ethical approval.

### Data Source and Search Strategy

To capture all relevant clinical and economic evidence, we conducted a comprehensive systematic search of MEDLINE (via PubMed), Embase, and the Cochrane Library from database inception to November 12, 2025. We did not apply any language restrictions. We deployed parallel search strategies within these identical databases to identify model-based economic evaluations. We iteratively refined our search strings using both Medical Subject Headings (MeSH) and free-text keywords (Table S1) and manually screened the reference lists of the included studies and relevant review articles to ensure exhaustiveness. Grey literature (refers to research that has not been published in traditional peer reviewed journals) was not searched for. After exporting all citations to Zotero and removing duplicates, 2 independent reviewers screened the titles and abstracts, followed by full-text assessments of potentially eligible articles. Discrepancies at any stage were resolved through discussion and consensus agreement.

### Eligibility Criteria

We selected studies based on strictly predefined eligibility criteria. We included clinical studies of any design (randomized controlled trials and observational cohorts) that evaluated adults with iron deficiency receiving intravenous iron therapy. The primary intervention was ferric carboxymaltose (FCM) directly compared with ferric derisomaltose (FDI).

To qualify for the primary quantitative meta-analysis, studies had to report extractable event-based incidence data for biochemical hypophosphatemia specifically within the 35- to 42-day post-infusion window. We excluded studies reporting outcomes solely as point-prevalence estimates, duration-based metrics, or summary measures lacking numerator-denominator data from quantitative pooling, although we retained them for narrative synthesis. Secondary outcomes included the incidence of severe hypophosphatemia, persistence at late follow-up (≥5-8 weeks), symptom burden, quality-of-life measures, and economic outcomes. Model-based economic evaluations (e.g., cost-utility analyses) were narratively synthesized due to substantial methodological heterogeneity and their reliance on shared clinical trial populations, thereby excluding them from the quantitative meta-analysis. Non-original research articles, editorials, and commentaries were excluded.

### Data Extraction and Quality Assessment

Two independent reviewers extracted the data using a standardized template. The extracted variables included publication details, intervention specifics (iron formulation and cumulative dose), baseline patient characteristics, and all primary and secondary outcomes. A third author adjudicated any unresolved conflicts in data extraction.

We assessed the methodological quality and risk of bias using validated tools tailored to each study design. Randomized controlled trials were evaluated using the Cochrane Risk of Bias 2 (RoB 2) tool, while observational studies were assessed using the Newcastle-Ottawa Scale (NOS). To ensure methodological rigor in our economic narrative synthesis, we evaluated economic studies using the Drummond checklist for methodological quality and the Consolidated Health Economic Evaluation Reporting Standards (CHEERS) tool for reporting quality. Discrepancies in the quality assessment were resolved through discussion.

### Statistical Analysis and Data Synthesis

All quantitative statistical analyses were conducted using Review Manager (RevMan, version 5.4). For our primary dichotomous outcome, we generated pooled risk ratios (RRs) with 95% confidence intervals (CIs). To account for the expected clinical heterogeneity across study populations, we employed a random-effects model utilizing generic inverse variance weighting. We visually represented the pooled treatment effects using forest plots. Statistical heterogeneity was assessed using Higgins *I*^2^ statistic, with values of 25% to 50%, 50% to 75%, and >75% denoting mild, moderate, and substantial heterogeneity, respectively. We planned sensitivity and subgroup analyses a priori to explore the potential sources of heterogeneity, contingent upon sufficient data availability. A 2-sided *P*-value of <.05 was considered statistically significant.

### Reporting Guideline

We used the PRISMA 2020 reporting guideline^16^ to draft this manuscript, and the PRISMA 2020 reporting checklist^17^ when editing, included in supplementary materials.

## Results

### Search Results and Study Quality

Our systematic search from database inception to November 12, 2025, yielded 411 records from PubMed, Embase, and Cochrane databases. Following rigorous screening and removal of duplicates, 21 studies were retained for the final synthesis (Figure 1). The quantitative meta-analysis comprised 4 randomized comparisons derived from 3 published randomized controlled trials (RCTs), whereas the narrative synthesis incorporated 18 studies (6 observational cohorts and 12 economic evaluations). The detailed baseline characteristics are outlined in Tables 1 and 2 (economic evaluation studies).

**Figure 1.**
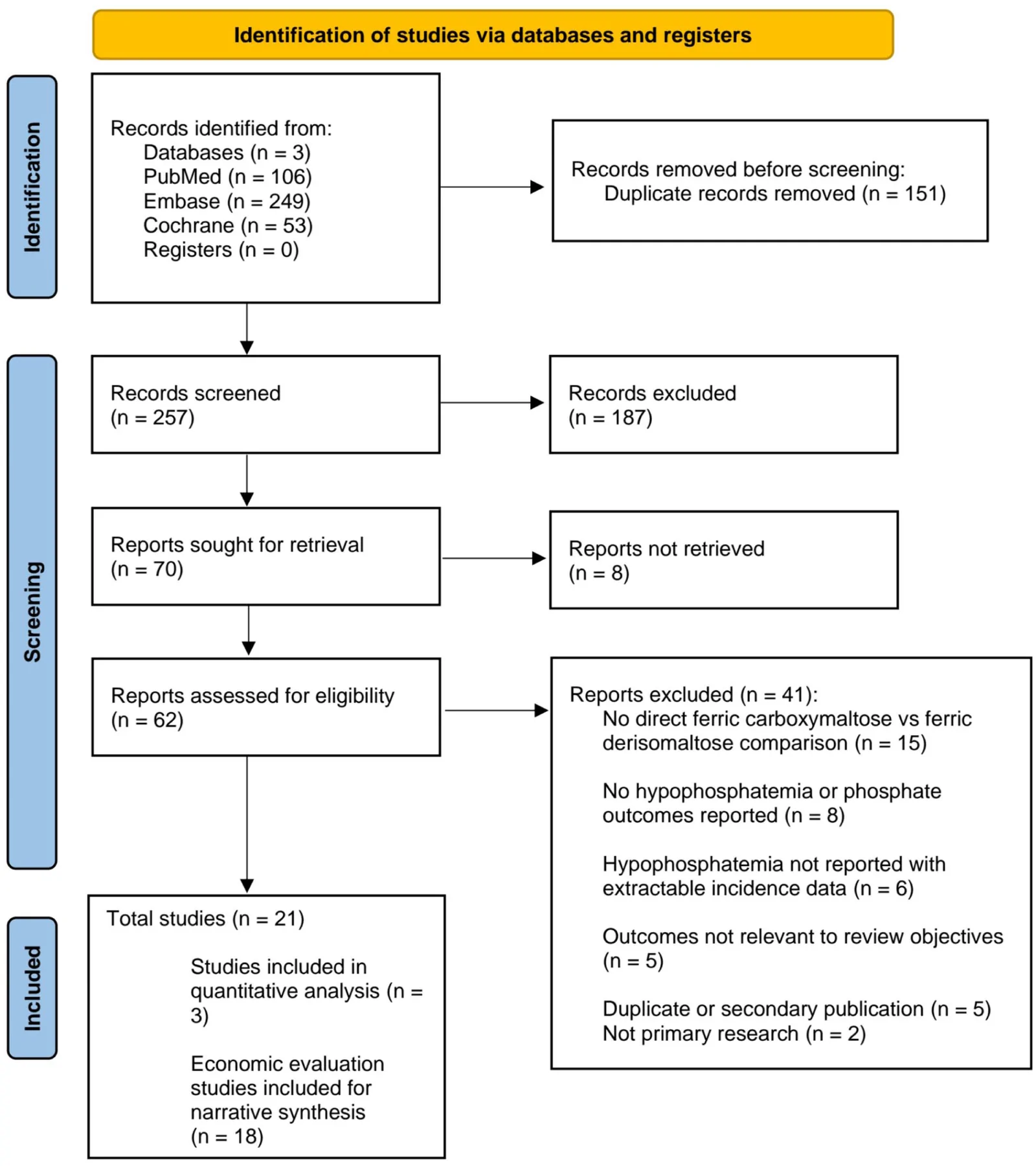
PRISMA flow diagram.

**Table 1.** Baseline Characteristics.

First author (year) Trial name			Schaefer et al. (2016)	Bager et al. (2016)	Detlie et al. (2019)	Wolf et al. (2020)		Emrich et al. (2020) HOMe aFers	Detlie et al. (2021)	Zoller et al. (2022) PHOSPHARE IBD	Marti et al. (2023)	Kassianides et al. (2020) ExplorIRON CKD
						Trial A	Trial B					
Journal			*PLOS ONE*	*British Journal of Pharmacology*	*WILEY Library*	*JAMA*		*BMC Medicine Journal*	*World Journal of Gastroenterology*	*GUT Journal*	*Journal of Personalized Medicine (JPM)*	*BMC Nephrology journal*
Country			Austria	Denmark	Norway	USA		Europe	Norway	Europe	Poland	United Kingdom
Study design			Retrospective Observational	Retrospective Observational	Prospective Observational	RCT		RCT	Prospective Observational	RCT	Prospective Observational	RCT
Study period			1st Jan. 2002 to 10th Oct. 2015	Nov. 2011 to April 2014	1Feb.2017 to 1July 2018	Oct. 2017 to June 2018	Oct. 2017 to May 2018	Aug. 2016 to Aug.2018	1st Feb. 2017 to 1st July 2018	23 May 2018 to 25 May 2020	2016 to 2020	Mar. 2020 to July 2021
Total patients, n			81	231^b^	106	123	119	26	106	97	56	26
FCM, n (%)			55 (68)	125 (54.1)	52 (50.5)	60 (48.8)	57 (47.9)	13 (50)	52 (49.6)	49 (50.5)	12 (21.4)	12 (46.6)
FDI, n (%)			26 (32.0)	67 (29.0)	54 (41.5)	63 (51.2)	62 (52.1)	13 (50)	54 (50.9)	48 (49.5)	44 (78.6)	14 (53.8)
Follow up time points			Variable	Week 2, 5, 10	Week 2, Week 6	Days 7-35		Day 7 and 35	Week 2 and 6	Day 35, Day 70	Week 1, 4, 12	Baseline, ~1 mo, ~2 mo
Dosage, mg	FCM	Initial dosage	NR	1000 (500-2500)^a^	1000	750 (Day 0)		1000	1000	1000 (Day 0)	NR	1000 (Day 0)
		Repeat dosage				750 (Day 7)				500 (Day 35) in 69.4%^c^		500/1000 (1 mo)^d^
	FDI	Initial dosage	NR		1000	1000 (Day 0)		1000	1000	1000 (Day 0)	NR	1000 (Day 0)
		Repeat dosage								500 (Day 35 in 66.7%^c^		500/1000 (1 mo)^d^
Weight, kg	FCM		NR	NR	NR	77.4 (20.2)	84.2 (20.1)	NR	NR	80.6 (16.6)	NR	NR
	FDI		NR	NR	NR	80.6 (16.6)	90.1 (29.2)	NR	NR	79.8 (15.4)	NR	NR
Age, years	FCM		49 (38.5-68.5)^a^	41 (18-87)^a^	40.6 (14.1)	46.3 (11.6)	43.1 (11.5)	40 (10 )	40.6 (14.1)	41.9 (14.7)	CD = 38 (14.99); UC = 35.35 (13.51)	73.2 (10.8)
	FDI				39.5 (13.5)	43.9 (10.4)	42.2 (12.9)	34 (11)	39.5 (13.5)	42.3 (14.1)		63.4 (12.2)
Sex	Male n (%)	FCM	Nil	74 (32.0)	23 (44.2)	3 (5.0)	3 (5.3)	Nil	23 (44.2)	24 (49.0)	32 (57.1)	9 (75.0)
		FDI	Nil		29 (53.7)	2 (3.2)	4 (6.5)	Nil	29 (53.7)	22 (45.8)		8 (57.1)
	Female n (%)	FCM	55 (68)	156 (67.5)	29 (55.8)	57 (95.0)	54 (94.7)	13 (50)	29 (55.8)	25 (51.0)	24 (42.9)	3 (25.0)
		FDI	26 (32.0)		25 (46.3)	61 (96.8)	58 (93.5)	13 (50)	25 (46.3)	26 (54.2)		6 (42.8)
Underlying diagnosis	CD		17 (21)	101 (43.7)	47 (44.3)	Nil	Nil	Nil	47 (44.3)	38 (39.2)	32 (57.1)	Nil
	UC		19 (23.5)	43 (18.6)	59 (55.7)	Nil	Nil	Nil	59 (55.7)	59 (60.8)	24 (42.9)	Nil
	Celiac disease		Nil	9 (3.9)	Nil	Nil	Nil	Nil	Nil	Nil	Nil	Nil
	IDA		Nil	17 (7.4)	Nil	123	119	26	Nil	Nil	Nil	Nil
	Anemia-others		Nil	23 (10.0)	Nil	Nil	Nil	Nil	Nil	Nil	Nil	nil
	Non-dialysis dependent CKD		Nil	Nil	Nil	Nil	Nil	Nil	Nil	Nil	Nil	26
	Occult blood loss		20 (24.7)	Nil	Nil	Nil	nil	nil	nil	nil	nil	nil
	Others		25 (30.9)	38 (16.4)	Nil	Nil	nil	nil	nil	nil	nil	nil
Baseline hemoglobin, g/dL	FCM		9.9 (2.2)^e^	11.5 (10.3-12.7)^a^	12.4 (1.6)	9.6 (1.3)	9.3 (1.4)	10.1 (1.4)	12.4 (1.6)	10.4 (1.4)^e^	11.3 (2.8)^a^	10.1 (1.5)^e^
	FDI				11.6 (1.8)	9.8 (1.3)	9.6 (1.2)	10.7 (1.2)	11.6 (1.8)	10.5 (1.5)^e^		9.9 (1.2)^e^
Post treatment hemoglobin, g/dL	FCM		11.9 (1.8)^e^	NR	13.3 (1.4)	12.5 (1.1)	12.3 (1.2)	NR	13.3 (1.4)	NR	13.0 (2.5)^a^	10.6 (1.4)^e^
	FDI		10.9 (1.8)^e^	NR	13.4 (1.5)	12.1 (1.0)	11.8 (1.4)	NR	13.4 (1.5)	NR		10.4 (1.3)^e^
Baseline serum phosphate, mg/dL	FCM		3.0 (2.8-3.5)^a,f^	3.2 (2.9-3.6)^a^	3.3 (0.5)^f^	3.3. (0.5)	3.3 (0.5)	3.2 (0.4 )	3.3 (0.5)^f^	3.5 (0.6)	3.4 (0.6)^a^	3.7 (3.3-4.3)^a f^
	FDI				3.5 (0.5)^f^	3.3 (0.6)	3.4 (0.5)	3.6 (0.6)	3.5 (0.5)^f^	3.5 (0.6)		4.0 (3.6-4.9)^a f^
Post treatment serum phosphate, mg/dL	FCM		2.5 (1.0)^f^	NR	3.1 (0.9)^f^	2.4 (0.9)	2.3 (0.9)	2.5 (0.8)	3.1 (0.9)^f^	NR	3.0 (1.0)^a^	3.5 (2.9-4.1)^a f^
	FDI		3.4 (0.81)^f^		3.5 (0.6)^f^	3.5 (0.6)	3.5 (0.5)	3.2 (0.6)	3.5 (0.6)^f^	NR		3.6 (3.3-4.9)^a f^

Abbreviations: FCM, ferric carboxymaltose; FDI, ferric derisomaltose; CD, crohn’s disease; UC, ulcerative colitis; IDA, iron deficiency anemia; CKD, chronic kidney disease.

Continuous variables are reported as mean (SD) unless otherwise indicated.

a Denote median (IQR). Categorical variables are reported as n (%).

b 39 patients out of 231 received both FCM and FDI treatment.

c Repeat dosing at Day 35 was protocol defined and conditional. Percentages indicate the proportion of patients who received any repeat infusion. Repeat dose amounts varied according to individual iron requirements, with some patients receiving higher repeat doses (up to 1000 mg).

d Based on weight and hematinic profile.

e Hemoglobin values originally reported in g/L were converted to g/dL for consistency.

f Serum phosphate values originally reported in mmol/L or were converted to mg/dL for consistency.

**Table 2.** Secondary Clinical Outcomes.

Outcome category	Study	Design	Population	Follow up	FCM n/N (%)	FDI/comparator n/N (%)	Effect measure	Effect estimate (95% CI)
Presence of hypophosphatemia (≥5-8 wk)								
	Schaefer 2016	Observational	Iron deficiency	≥8 wk	13/17		NA	NA
	Bager 2016	Observational (infusion series)	Iron deficiency	Week 10	6/165	1/165	FCM, POR	3.6 (0.4-30.4)
	Detlie 2019	Observational	IBD-IDA	Week 6	11/52(21.5%)	2/54(3.7%)	NA	NA
	Zoller 2022	RCT	IBD-IDA	Week 10	2/43(4.7%)	0/44	Risk difference	−4.7 (−10.9-1.6)
Incidence of severe hypophosphatemia								
	Detlie 2019	Observational	IBD-IDA	Week 2	1/52(2.0%)	0/54	NA	NA
	Wolf 2020	RCT	IDA	≤35 d	14/122(11.3%)	0/123	NA	NA
Symptomatic hypophosphatemia								
	PHOSPHARE-IBD	RCT	IBD-IDA	Day 35-49	NA	NA	LS mean difference (FACIT-Fatigue)	+5-6 points with FDI
	Detlie 2021	Extension-observational	IBD-IDA	Baseline	NA	NA	NA	NA
Quality of life								
	Detlie 2021	Extension-observational	IBD-IDA	Week 2-6	NA	NA	NA	NA
Economic evaluation^a^								
	Zoller 2024	Cost-utility analysis	IBD-IDA	10 y	NA	NA	ICUR	FDI dominant; +0.157 QALYs
	Iqbal 2024	Cost-utility analysis	IBD-IDA	5 y	NA	NA	ICUR	FDI dominant; +0.075 QALYs
	Zhang 2024	Cost-utility analysis	IBD-IDA	5 y	NA	NA	ICUR	FDI dominant; +0.070 QALYs
	Lindgren 2025	Cost-utility analysis	IBD-IDA	5 y	NA	NA	ICUR	FDI dominant; +0.076 QALYs
	Detlie 2025	Cost-utility analysis	IBD-IDA	5 y	NA	NA	ICUR	FDI dominant; +0.071 QALYs
	Pollock 2020	Cost-effectiveness analysis	IDA	Per treatment course	NA	NA	Incremental response rate	FDI dominant by 9%
	Aksan 2021	Cost-effectiveness analysis	IBD-IDA	Per treatment course	NA	NA	incremental response rate	FCM dominant by 6%
	Ramirez 2022	Cost-effectiveness analysis	IBD-IDA	Per treatment course	NA	NA	Incremental response rate	FCM dominant by 7%
	Pollock 2021	Budget impact analysis	IBD-IDA	5 y	NA	NA	Infusion per patient	0.39 fewer with FDI
	Loughnane 2019-2020	Resource impact analysis	IDA	Per treatment course	NA	NA	Additional patients treated and net annual clinical balance	+121 patients/year and +€6,408/year in favor of FDI
	Bhandari 2011	Cost-minimization analysis	Iron deficiency	Per treatment course	NA	NA	Mean cost saving-based on dose	1.74%-17.65% saved with FDI

Abbreviations: CI, confidence interval; FCM, ferric carboxymaltose; FDI, ferric derisomaltose; IBD, inflammatory bowel disease; ICUR, incremental cost utilization ratio; IDA, iron deficiency anemia; LS, least square; POR, prevalence odds ratio; QALY, quality adjusted life year; RCT, randomized controlled trial.

a Economic outcomes were derived from model-based analyses using published clinical, cost, and resource utilization inputs; no primary patient level economic data were collected within the included studies.

Overall, the synthesized clinical evidence demonstrated a high methodological integrity. The randomized trials, encompassing 364 iron-deficient patients (178 randomized to FCM and 186 to FDI), predominantly exhibited a low risk of bias or only some concerns according to the RoB 2 tool (Figure 2, Table S3). Similarly, the observational cohorts achieved strong NOS scores, ranging from 7 to 9 out of 9 stars (Figure 3, Table S4). The core methodological strengths of these clinical studies included the use of objective biochemical endpoints, standardized intravenous iron protocols, and follow-up durations sufficiently extended to capture both the onset and resolution of phosphate derangements. Despite limitations, such as partial blinding in some trials and non-randomized allocation in cohorts, the consistent application of rigorous biochemical definitions strongly supports the internal validity of these findings.

**Figure 2.**
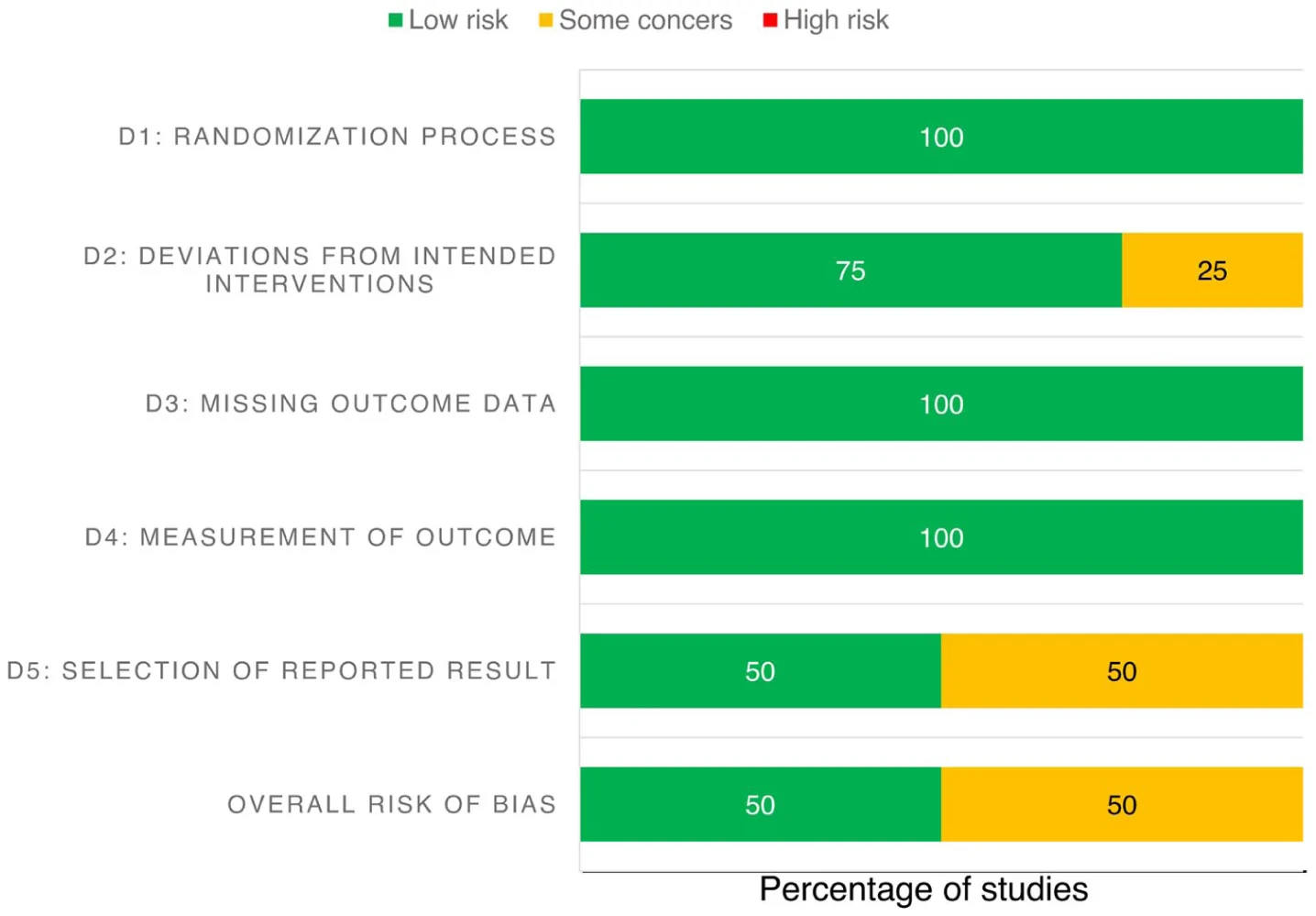
Summary of quality assessment for RCTs.

**Figure 3.**
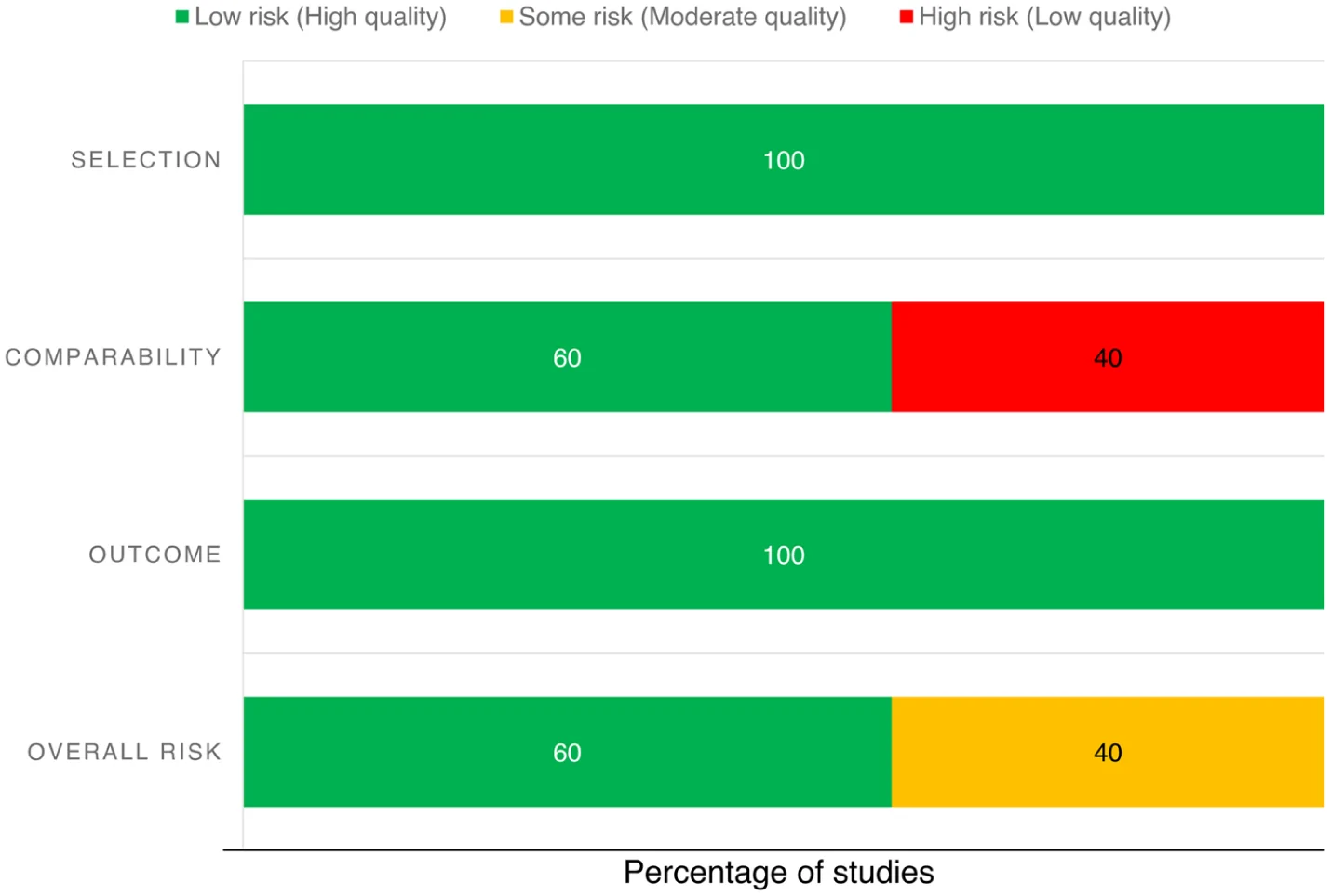
Summary of quality assessment for observational studies.

The 12 economic evaluations also exhibited moderate-to-high methodological quality based on the Drummond checklist and good reporting quality according to the CHEERS criteria (Figure 4, Table S5). While most models successfully utilized high-quality RCT evidence, clearly defined perspectives, and robust sensitivity analyses, several were limited by industry funding or the critical omission of societal costs related to long-term skeletal complications, such as osteomalacia.

**Figure 4.**
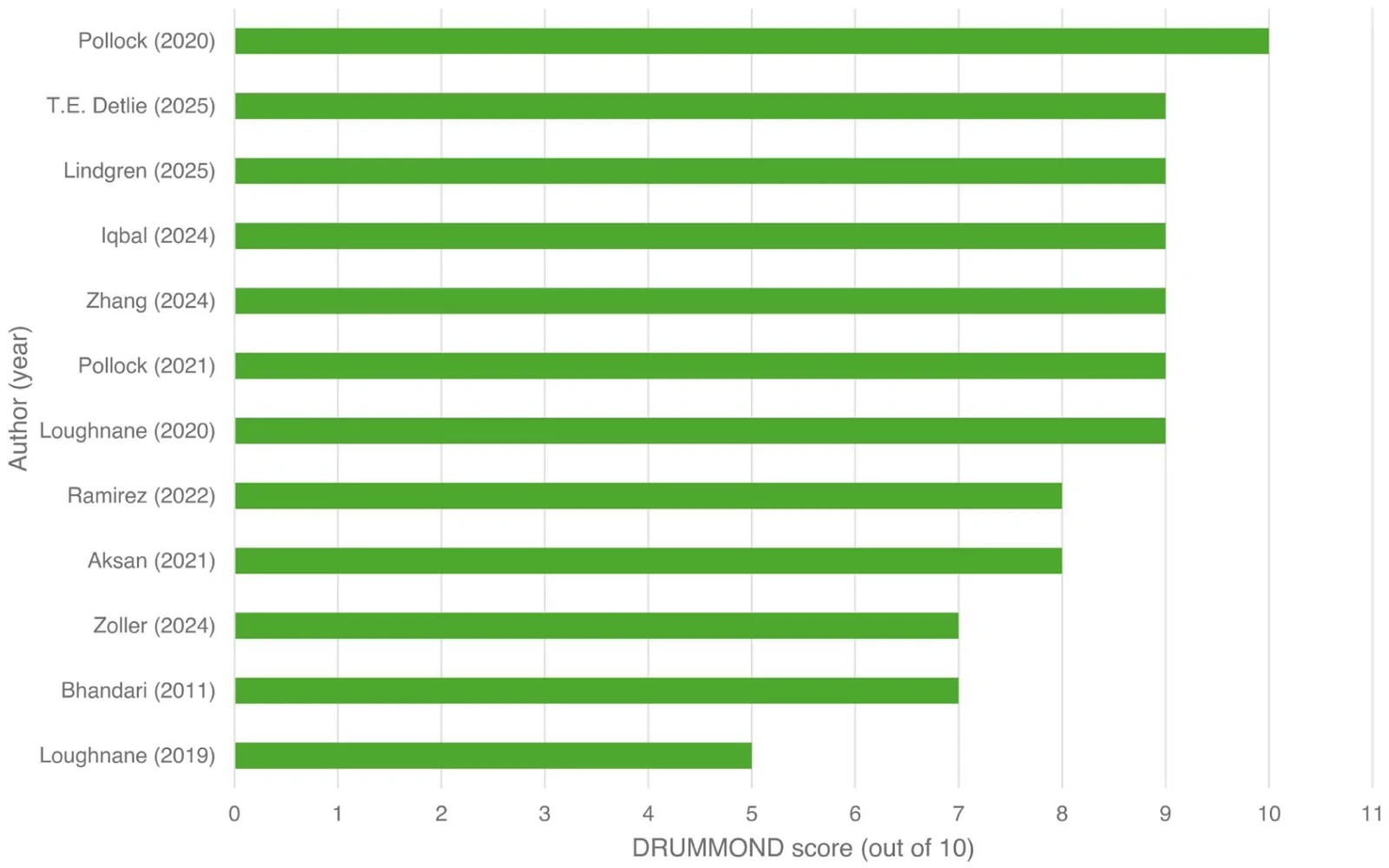
Quality assessment of economic evaluation studies.

### Primary Outcome: Incidence of Hypophosphatemia (35-42 Days)

In our pooled analysis of 364 participants across 4 randomized comparisons, FCM was associated with a significantly higher risk of incident hypophosphatemia within 35 to 42 days than FDI. Specifically, FCM conferred an increased risk of more than 8-fold (RR 8.37, 95% CI 5.10-13.75; *P* < .00001; Figure 5). These randomized findings were corroborated by an independent retrospective observational cohort that reported arm-specific incidence: biochemical hypophosphatemia (<0.8 mmol/L or <2.5 mg/dL) occurred in 45.5% (25/55) of FCM-treated patients, contrasting sharply with only 3.8% (1/26) in the FDI cohort. Because the number of eligible studies providing extractable outcome data was limited, we deferred a priori sensitivity and subgroup analyses to preserve the statistical reliability.

**Figure 5.**
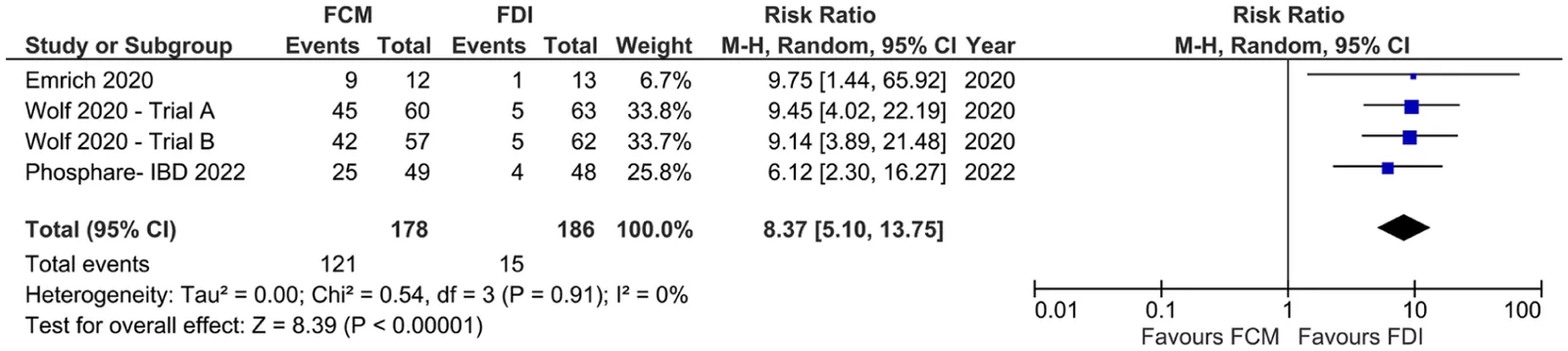
Effects of ferric carboxymaltose versus ferric derisomaltose on the incidence of hypophosphataemia within 35 to 42 days. Forest plot showing the effect of ferric carboxymaltose (FCM) compared with ferric derisomaltose (FDI) on the development of hypophosphataemia within 35 to 42 days in patients with iron deficiency. FCM significantly increased the risk of hypophosphatemia compared to that of FDI.

## Secondary Clinical Outcomes

### Severity and Persistence

Owing to the inherent heterogeneity in outcome definitions, dosing strategies, and follow-up intervals, we synthesized all secondary outcomes narratively (Table 2).

#### Severity of Phosphate Depletion

When reported as a biochemical safety outcome, severe hypophosphatemia occurred exclusively in the FCM treatment arms. In 1 RCT, severe hypophosphatemia (<1 mg/dL) was observed in 11.3% of FCM patients compared with 0% of FDI patients (*P* < .001). A similar formulation-specific disparity was observed in an inflammatory bowel disease (IBD) cohort at week 2 (2.0% FCM vs. 0% FDI), although no severe cases were observed in either group by week 6.

#### Persistence at Late Follow-up (≥5-8 Weeks)

Hypophosphatemia frequently persisted beyond the acute phase in FCM-treated patients. In randomized data, 21.6% of the FCM cohort remained hypophosphatemic at the 6-week visit, compared with only 3.7% of the FDI cohort. Extended randomized follow-up data up to day 70 confirmed this chronicity, with 4.7% (2/43) of FCM patients remaining hypophosphatemic compared with zero persistent cases in the FDI group. Observational infusion series mirrored these findings, documenting persistent phosphate abnormalities in substantial patient subsets (e.g., 7/165 patients at week 10 and 13/17 patients with extended monitoring beyond 8 weeks). Furthermore, an exploratory study in patients with CKD demonstrated that mean serum phosphate levels remained reduced for approximately 2 months (−13.2% change from baseline) in the FCM group, whereas it recovered (+9.2% change) in the FDI group.

### Patient-Reported and Health Economic Outcomes

#### Symptomatic Burden and Quality of Life (QoL)

While intravenous iron generally improved fatigue across all patients, the magnitude of symptomatic benefit appeared blunted by phosphate depletion. In a randomized follow-up, FDI elicited greater improvements in fatigue scores on Days 35 and 49 compared with FCM; crucially, this greater fatigue improvement was statistically associated with smaller declines in serum phosphate levels. Mechanistic extension studies using visual analog scales indicated that patients with hypophosphatemia had lower baseline scores across multiple domains (including fatigue and bone pain), although subsequent symptom changes at weeks 2 and 6 were small and did not consistently separate hypophosphatemic from non-hypophosphatemic patients. Similarly, the overall QoL assessed using the SF-36 questionnaire did not differ significantly between patients with and without hypophosphatemia at baseline or early follow-up.

#### Economic and Health-System Impact

The divergent safety and dosing profiles of these formulations directly translate into significant economic variations. Across multiple cost-utility analyses in IBD-IDA models, FDI consistently dominated FCM, yielding higher quality-adjusted life years (QALY gains of 0.070-0.157 over 5-10 years) alongside lower total costs. FDI remained dominant in all base cases and was cost-effective across various sensitivity analyses and willingness-to-pay thresholds. Although cost-effectiveness models based strictly on hematologic response yielded mixed dominance, with some alternative strategy models favoring FCM by margins of 6% to 9%, FDI demonstrated distinct operational advantages. Budget impact and resource models projected that FDI optimizes infusion center capacity by treating more patients under fixed constraints, primarily driven by its ability to safely deliver higher cumulative iron doses in fewer administrations. Consequently, cost-minimization analyses demonstrated lower administration costs with FDI across clinically relevant doses, yielding modeled savings ranging from 1.7% at lower doses to 17.6% at higher total iron requirements.

## Discussion

In this systematic review and meta-analysis of adults receiving intravenous iron for IDA, FCM was associated with a significantly higher incidence of acute hypophosphatemia within 35 to 42 days than FDI. Across strictly randomized head-to-head comparisons, FCM conferred an over 8-fold increased risk of this metabolic derangement (RR 8.37), driven by a consistent, formulation-specific effect completely devoid of statistical heterogeneity.^11,18^

This robust quantitative finding is consistent with established mechanistic data implicating the FGF23 axis.^7^ While iron deficiency itself increases FGF23 transcription, FCM administration uniquely blocks the cleavage of this hormone into inactive fragments, resulting in a massive accumulation of active, intact FGF23.^6,7^ Conversely, FDI induces only minimal or transient changes in iFGF23 cleavage, which elegantly explains the negligible incidence of hypophosphatemia and rapid phosphate normalization universally observed in the FDI trial arm.^11,18^ By restricting our primary synthesis to randomized head-to-head comparisons, we eliminate the confounding variables (such as underlying renal function or baseline inflammation) that plagued earlier observational cohorts, thereby strengthening causal inference and strongly supporting that severe hypophosphatemia is a formulation-dependent adverse outcome, not an inevitable IV iron class effect.^13,18^

The clinical significance of this biochemical divergence is immense. When explicitly reported, severe hypophosphatemia (<1 mg/dL) occurred predominantly in FCM-treated patients and was completely absent among those receiving FDI.^11,18^ Even more concerning is the chronicity of this depletion: multiple included studies documented hypophosphatemia persisting well beyond 5 to 8 weeks following FCM exposure.^13,18^ This delayed recovery timeline critically intersects with recent systematic reviews and regulatory pharmacovigilance reports documenting severe skeletal consequences, such as widespread osteomalacia and atypical insufficiency fractures, following repeated FCM administration.^8,10^

Furthermore, while intravenous iron generally improves baseline fatigue, this symptomatic benefit is significantly attenuated in patients experiencing phosphate declines.^11,18^ This insidious dissociation between hematologic correction and patient-perceived recovery highlights a critical clinical pitfall: iatrogenic phosphate depletion actively undermines the primary therapeutic goals of iron repletion.^8^ Symptomatic hypophosphatemia is not a benign laboratory artifact; it is a profound clinical harm.^9^

Beyond individual patient harm, this formulation-specific safety signal exerts a measurable toll on healthcare system resources. Across published cost-utility and service delivery analyses conducted in different healthcare systems, FDI generally demonstrated greater economic efficiency than FCM, although these findings were based on different economic models and healthcare assumptions. This is primarily driven by its capacity to safely deliver higher cumulative iron doses in fewer infusions, drastically reducing the infusion unit time and resource utilization. Crucially, current economic models routinely underestimate the true cost of FCM by failing to incorporate the downstream expenditures mandated by regulatory safety communications, namely, the costs of prolonged biochemical monitoring, phosphate supplementation, and long-term multidisciplinary management of osteomalacia.^8,10^ Factoring in these downstream costs may further improve the relative cost-effectiveness of FDI. However, these economic findings should be interpreted with caution, as cost-effectiveness is influenced by local clinical practice, healthcare reimbursement structures, resource availability, and model assumptions. Consequently, the relative economic value of FDI and FCM may differ across healthcare settings.

Taken together, these findings support consideration of formulation-specific metabolic safety alongside local economic and healthcare system factors when selecting intravenous iron therapy. For adults requiring intravenous iron, particularly those anticipating repeated infusions (e.g., IBD or abnormal uterine bleeding) or harboring baseline risk factors for bone disease, the preferential selection of FDI effectively eliminates a severe metabolic safety risk while preserving hematologic efficacy.^11,18^ If FCM must be utilized, clinicians should implement baseline phosphate assessments and extended post-infusion monitoring, especially for symptomatic individuals. At the policy level, hypophosphatemia must be formally incorporated into institutional comparative value assessments (P&T committees) for intravenous iron therapy. Future research should prioritize long-term, prospective trial designs utilizing bone biopsies or advanced imaging to definitively quantify the incidence of osteomalacia and fracture risk.^8,10^

## Limitations

This study has several limitations. First, the restricted number of eligible randomized trials limited the statistical precision for rare events and precluded reliable subgroup analyses. Furthermore, the lack of individual patient data limited our assessment of critical baseline effect modifiers such as vitamin D status and cumulative dosing. Beyond the primary endpoint, extreme heterogeneity in the reporting of secondary outcomes necessitated narrative synthesis. Finally, robust data regarding long-term skeletal consequences are largely derived from case reports and systematic reviews of observational data rather than controlled, prospective trials.

## Conclusion

Among adults receiving intravenous iron for IDA, ferric carboxymaltose provokes a substantially higher incidence of hypophosphatemia than ferric derisomaltose, with overwhelming evidence demonstrating greater clinical severity, prolonged persistence, and attenuated quality of life benefits. These findings underscore the importance of integrating formulation-specific metabolic safety profiles into both frontline clinical selection and institutional economic pathways for IV iron therapy.

## Supplemental Material

sj-docx-1-hpx-10.1177_00185787261485528 – Supplemental material for Time Course and Comparative Risk of Hypophosphatemia After Ferric Carboxymaltose versus Ferric Derisomaltose: A Systematic Review, Meta-analysis, and Clinical Cost Considerations Supplemental material, sj-docx-1-hpx-10.1177_00185787261485528 for Time Course and Comparative Risk of Hypophosphatemia After Ferric Carboxymaltose versus Ferric Derisomaltose: A Systematic Review, Meta-analysis, and Clinical Cost Considerations by Vidhita Dangre, Mohd Rafiuddin, Krithika Sriram, Iqra Aslam Siddiqui, Hadia Fahim, Shanthakumar Sakthivel Murugan, Federica Copeta, Noor Ul Ain Fatima, Belinda Eze and Swostika Pokhrel in Hospital Pharmacy

## Data Availability

All data analyzed in this systematic review and meta-analysis were derived from previously published studies. The extracted dataset and supplementary materials supporting the findings of this study will be provided as supplementary materials alongside the manuscript.
