# Supplementary material for: Time Course and Comparative Risk of Hypophosphatemia After Ferric Carboxymaltose versus Ferric Derisomaltose: A Systematic Review, Meta-analysis, and Clinical Cost Considerations
Source: Hosp Pharm. 2026 Oct 24:00185787261485528. Online ahead of print. doi: 10.1177/00185787261485528 (PMC13696579; doi:10.1177/00185787261485528)
Supplement: Supplementary file 1 [file sj-docx-1-hpx-10.1177_00185787261485528.docx]

# Supplementary Material

**Supplementary Material 1:** PICO Framework Diagram

**Supplementary Material 2:** Search Strategy

**Supplementary Material 3:** Baseline Characteristics of economic evaluation studies

**Supplementary Material 3:** Risk of Bias Assessment

**Graphical Abstract**

**Supplementary Material 1:** PICO Framework Diagram

**Online Figure S1:** Population, Intervention, Comparison, and Outcome (PICO) framework

**Supplementary Material 2:** Search Strategy

**Online Table S1:** Search strategy

| **Database** | **Search terms** | **No. of articles found** |
| --- | --- | --- |
| **Pubmed** | (“iron deficiency” OR “iron deficiency anemia” OR “iron-deficiency anemia” OR “IDA” OR “iron-deficient” OR “anemia” OR “anaemia” OR “Iron-depleted” OR “depleted iron stores” OR “hypophosphatemia”)  AND  (“intravenous iron” OR “IV iron” OR “parenteral iron” OR “iron infusion” OR “iron replacement therapy”)  AND  (“ferric carboxymaltose” OR “FCM” OR “ferinject”)  AND  (“ferric derisomaltose” OR “FDI” OR “iron isomaltoside” OR “monofer”) | 106 |
| **Embase** | (“iron deficiency” OR “iron deficiency anemia” OR “iron-deficiency anemia” OR “IDA” OR “iron-deficient” OR “anemia” OR “anaemia” OR “Iron-depleted” OR “depleted iron stores” OR “hypophosphatemia”)  AND  (“intravenous iron” OR “IV iron” OR “parenteral iron” OR “iron infusion” OR “iron replacement therapy”)  AND  (“ferric carboxymaltose” OR “FCM” OR “ferinject”)  AND  (“ferric derisomaltose” OR “FDI” OR “iron isomaltoside” OR “monofer”) | 249 |
| **Cochrane CENTRAL** | (“iron deficiency” OR “iron deficiency anemia” OR “iron-deficiency anemia” OR “IDA” OR “iron-deficient” OR “anemia” OR “anaemia” OR “Iron-depleted” OR “depleted iron stores” OR “hypophosphatemia”)  AND  (“intravenous iron” OR “IV iron” OR “parenteral iron” OR “iron infusion” OR “iron replacement therapy”)  AND  (“ferric carboxymaltose” OR “FCM” OR “ferinject”)  AND  (“ferric derisomaltose” OR “FDI” OR “iron isomaltoside” OR “monofer”) | 53 |

**Supplementary Material 3:** Baseline Characteristics of economic evaluation studies

**Online Table S2:** Baseline Characteristics of economic evaluation papers

| Author/Year | Economic Study design | Journal | Country/ Region | Model type | Perspective | Population type | Time horizon, years | Currency (Price year) | Discounting-annual discount rate, % | Primary Economic Outcome |
| --- | --- | --- | --- | --- | --- | --- | --- | --- | --- | --- |
| Zoller et al. (2024) | Cost-utility analysis | Value in Health journal | UK | Patient-level simulation | UK National healthcare system (NHS) | IBD-ID | 10 | GBP(NR) | 3.5 | ICUR(QALYs) |
| Iqbal et al.(2024) | Cost-utility  Analysis | Journal of Medical Economics | England | Patient-level simulation | Department of Health and Social Care (DHSC) | IBD‑IDA | 5 | GBP(2022) | 3.5 | ICUR(QALYs) |
| Zhang et al.(2024) | Cost-utility  analysis | Springer Nature Link | China | Patient level simulation | Chinese healthcare system | IBD‑IDA | 5 | CNY(2023) | 5 | ICUR(QALYs) |
| Lindgren et al.(2025) | Cost-utility  analysis | Journal of Medical Economics (JME) | Sweden, Austria, Denmark, Germany, UK | Micro simulation | Swedish national healthcare payer | IBD‑IDA | 5 | SEK(2024) | 3 | ICUR(QALYs) |
| Detlie et al.(2025) | Cost-utility  analysis | Journal of Medical Economics | Norway | Patient level simulation | Norwegian national payer | IBD‑IDA | 5 | NOK(2024) | 4 | ICUR(QALYs) |
| Pollock et al.(2020) | Cost-Effectiveness analysis | Journal of Medical Economics (JME) | UK | Patient level simulation | UK healthcare payer | IDA | Per Treatment course | GBP(2019-20) | nil | Incremental cost per responder |
| Aksan et al.(2021) | Cost-Effectiveness analysis | Dovepress journal: ClinicoEconomics and Outcomes Research | UK | Cohort model(Excel based) | NHS Hospital Trust | IBD‑IDA | Per Treatment course | GBP(2020) | nil | Incremental cost per responder |
| Antonio Ramirez et al.(2022) | Cost-Effectiveness analysis | Value in Health (Volume 25, Issue 12S, December 2022) | Sweden | Microsimulation | Swedish healthcare regions | IBD‑IDA | Per Treatment course | SEK(2021) | nil | Incremental cost per responder |
| Pollock et al. (2021) | Budget Impact Analysis | Dovepress journal: ClinicoEconomics and Outcomes Research | Norway, Sweden, Finland | Cohort level model | National healthcare system | IBD-IDA | 5 | EUR, NOK, SEK(NR) | nil | Budget impact; infusions |
| Loughnane et al.(2019 and 2020) | Resource Impact Analysis | Springer Nature Link (2020 full paper);Value in Health journal(2019 abstract) | Ireland | Resource impact model(Excel based) | Provider/clinic perspective | IDA (pre operative) | Per Treatment course | EUR(2019) | nil | Net clinic balance |
| Bhandari (2011) | Cost-minimization analysis | Dovepress journal: Therapeutics and Clinical Risk Management | UK | cost minimization model | UK healthcare system | IDA | Per Treatment course | GBP(NR) | nil | Mean cost difference |

**Supplementary Material 4:** Risk of Bias Assessment

**Online Table S3**: Quality Assessment for RCTs

| **Study ID / Citation** | **D1** | **D2** | **D3** | **D4** | **D5** | **Overall** |
| --- | --- | --- | --- | --- | --- | --- |
| Emrich et al. 2020 | 🟢 | 🟢 | 🟢 | 🟢 | 🟡 | 🟡 |
| Zoller et al. 2023 | 🟢 | 🟢 | 🟢 | 🟢 | 🟢 | 🟢 |
| Kassianides and Bhandari 2024 | 🟢 | 🟢 | 🟢 | 🟢 | 🟡 | 🟡 |
| Wolf et al. 2020 | 🟢 | 🟡 | 🟢 | 🟢 | 🟢 | 🟢 |
| **Legend:** \|; **D1: Bias arising from the randomization process** \|; **D2: Bias due to deviations from intended interventions** \|; **D3: Bias due to missing outcome data** \|; **D4: Bias in measurement of the outcome** \|; **D5: Bias in selection of the reported result** \|; **Overall: Overall risk of bias** \|; **Risk Level Key:** \|; 🟢 Low risk \|; 🟡 Some concerns \|; 🔴 High risk \| | | | | | | |

**Online Table S4:** Quality Assessment for Observational Studies

| **Study ID / Citation** | **Selection** | **Comparability** | **Outcome** | **Total Stars** | **Overall Risk** |
| --- | --- | --- | --- | --- | --- |
| Bager et al. 2017 | 🟢 | 🟢 | 🟢 | 9-9 | 🟢 |
| Delie et al. 2019 | 🟢 | 🔴 | 🟢 | 7-9 | 🟡 |
| Detlie TE et al. 2021 | 🟢 | 🔴 | 🟢 | 7-9 | 🟡 |
| Schaefer et al. 2016 | 🟢 | 🟢 | 🟢 | 9-9 | 🟢 |
| Tulewicz-Marti et al. 2023 | 🟢 | 🟢 | 🟢 | 9-9 | 🟢 |
| **Legend:**  Selection: Represents representativeness, selection of non-exposed, ascertainment of exposure, and outcome not present at start.  Comparability: Represents control for core and additional confounders.  Outcome: Represents assessment of outcome, length of follow-up, and adequacy of follow-up.  Total Stars: Cumulative score out of 9 possible stars.  **Risk Level Key:**  **🟢 Low risk (High quality)**  **🟡 Some risk (Moderate quality)**  **🔴 High risk (Low quality)** | | | | | |

**Online Table S5:** Quality Assessment of Economic Studies

| Author (Year) | Drummond Score (Methodology) | CHEERS Score (Reporting) | Key Strength | Key Weakness / Risk of Bias | HPP Inclusion in Model? |
| --- | --- | --- | --- | --- | --- |
| Bhandari (2011) | 7 / 10 | Moderate | First to model NHS nursing time savings. | Dated pharmacy pricing; simple CMA design. | No |
| Loughnane (2019) | 5 / 10 | Low (Abstract) | Captures real-world Irish labour costs. | Abstract only; lacks granular data. | No |
| Pollock (2020) | 10 / 10 | High | Patient-level simulation; high rigor. | Funded by FDI manufacturer. | Partial (Discussion only) |
| Loughnane (2020) | 9 / 10 | High | Focuses on clinic "throughput" capacity. | Specific to Irish pre-op setting. | Partial (Discussion only) |
| Pollock (2021) | 9 / 10 | High | Multi-country (Nordic) validation. | Budget impact (no QALYs). | Yes (Safety context) |
| Aksan (2021) | 8 / 10 | High | Uses NMA for efficacy (Hb response). | Funded by FCM manufacturer; HPP costs omitted. | No |
| Ramirez (2022) | 8 / 10 | High | Uses real-world regional tender prices. | Funded by FCM manufacturer; limited to Sweden. | No |
| Zhang (2024) | 9 / 10 | High (22/24 items) | well-defined population and objective, high quality data from an RCT (PHOSPHATE-IBD), full transparency in costs, and full disclose of funding. | Includes only the payer perspective, excluding costs of long-term complications, and declared conflict of interest as it is funded by the manufacturer of ferric derisomaltose (FDI) | Only short-term |
| Zoller (2024) | 7 / 10 | Moderate | First UK model to weight QALYs based on HPP risk. | Abstract only; limited data on sensitivity analysis. | Directly |
| Iqbal (2024) | 9 / 10 | High (22/24) | Well-defined population and objectives, high quality of evidence based on RCT, full transparency of costs and assumptions, inclusion of sensitivity analysis | Unmodeled long-term HPP complications, partial societal costs, industry funding, and limited generalisability outside England. | Only short-term |
| Lindgren (2025) | 9 / 10 | High (22/24) | clear objective and population, high quality evidence on RCT, transparency of costs, modelling and assumptions, performed sensitivity analysis | exclusion of long-term hypophosphatemia-related complications, partial inclusion of societal costs, industry funding (FDI) and results not applied outsides Sweden | Only short-term |
| T.E. Detlie (2025) | 9 / 10 | High | Integrates HPP incidence from PHOSPHARE into QALY model. | Funded by FDI manufacturer. | Directly |

**Graphical Abstract**

This meta-analysis examines the risk of low phosphate levels in adults with iron-deficiency anemia receiving intravenous iron, comparing two systems: ferric carboxymaltose (FCM) versus ferric derisomaltose (FDI). The analysis incorporated randomized head‑to‑head trials that used comparable methods to evaluate hypophosphatemia. The higher incidence occurred among patients receiving FCM, and the duration of the syndrome was more than 35-42 days, which implies that it was more dangerous. The effect seems to be selective to the formulation and associated with the fibroblast growth factor 23 (FGF23) elevation. Such an uptake can cause renal phosphate wasting and other metabolic problems. In contrast, FDI causes only a small change in phosphate levels and provides the same blood-related benefits, making it a better choice when keeping metabolism safe is important.

**Plain Language Summary:**

Iron deficiency anemia is commonly treated with intravenous iron when oral treatments are not suitable. Two commonly used formulations are ferric carboxymaltose (FCM) and ferric derisomaltose (FDI). While both are effective at improving iron levels, this study found that FCM is associated with a higher risk of low phosphate levels in the blood (hypophosphatemia), which can sometimes be severe or long lasting. In contrast, FDI showed a lower risk of this side effect while providing similar benefits in treating anemia. These findings suggest that the choice of intravenous iron formulation may have important implications for patient safety
